# Stakeholder perceptions of lethal means safety counseling: A qualitative systematic review

**DOI:** 10.3389/fpsyt.2022.993415

**Published:** 2022-10-20

**Authors:** Gabriela Kattan Khazanov, Shimrit Keddem, Katelin Hoskins, Karoline Myhre, Sarah Sullivan, Emily Mitchell, Brooke Dorsey Holliman, Sara J. Landes, Joseph Simonetti

**Affiliations:** ^1^Mental Illness Research, Education and Clinical Center, Corporal Michael J. Crescenz VA Medical Center, Philadelphia, PA, United States; ^2^Perelman School of Medicine, University of Pennsylvania, Philadelphia, PA, United States; ^3^Center for Health Equity Research and Promotion, Corporal Michael J. Crescenz VA Medical Center, Philadelphia PA, United States; ^4^James J. Peters VA Medical Center, Bronx, NY, United States; ^5^Department of Family Medicine, University of Colorado Anschutz School of Medicine, Aurora, CO, United States; ^6^Behavioral Health Quality Enhancement Research Initiative, Central Arkansas Veterans Healthcare System, Little Rock, AR, United States; ^7^Department of Psychiatry, University of Arkansas for Medical Sciences, Little Rock, AR, United States; ^8^VA Rocky Mountain Mental Illness Research, Education and Clinical Center for Suicide Prevention, Rocky Mountain Regional VA Medical Center, Aurora, CO, United States; ^9^Division of Hospital Medicine, University of Colorado Anschutz School of Medicine, Aurora, CO, United States

**Keywords:** suicide, lethal means, firearms, medications, qualitative, thematic synthesis, implementation, CFIR

## Abstract

**Introduction:**

Lethal means safety counseling (LMSC) is an evidence-based suicide prevention intervention during which providers encourage patients to limit their access to lethal means (e.g., firearms, medications). Despite agreement about the importance of LMSC, it is underutilized in clinical practice.

**Methods:**

To better understand the individual and contextual factors that influence LMSC and its implementation, we conducted a systematic review of qualitative studies examining stakeholder perceptions of the intervention. PubMed and PsycInfo were searched up to February 2021 using terms related to: (1) LMSC, firearms, or medications; (2) suicide, safety, or injury; and (3) qualitative methodology. Two coders used thematic synthesis to analyze findings from eligible papers, including developing a codebook and coding using an inductive and iterative approach (reliability *k* > 0.70). Confidence in review findings were evaluated using the Confidence in the Evidence from Reviews of Qualitative Research (CERQual) Approach. Subthemes were assigned to domains in the Consolidated Framework for Implementation Research.

**Findings:**

Of the 19 papers identified, 18 discussed LMSC for firearms and 1 focused exclusively on LMSC for medications. The firearm-related studies explored perspectives of a variety of stakeholders (patients, providers, members of the firearms community, healthcare leaders, and family members) across multiple settings (emergency departments, pediatric and adult primary care, and outpatient mental health). Seven overarching themes emerged, including the: (1) importance of firearms to owners’ identities and perceptions of ownership as a value and right, which can lead to perceived cultural tensions in clinical settings; (2) importance of patients understanding the context and rationale for LMSC; (3) value of providers showing cultural competency when discussing firearms; (4) influence of safety and risk beliefs on firearm behaviors; (5) need to navigate logistical concerns when implementing LMSC; (6) value of individualizing LMSC; (7) potential for trusted family members and friends to be involved in implementing LMSC.

**Conclusion:**

This synthesis of the qualitative literature informs clinical, operational, and research endeavors aimed at increasing the reach and effectiveness of LMSC. Future research should address the perspectives of individuals underrepresented in the literature (e.g., those from racial/ethnic minority groups) and further examine stakeholders’ perceptions of LMSC for medication. [-2pt]

**Systematic review registration:**

[https://www.crd.york.ac.uk/prospero/display_record.php?ID=CRD42021237515], identifier [CRD42021237515].

## Introduction

Suicide is the 10th leading cause of death in the United States (US), and suicide rates have increased by about 30% since 1999 (1). The majority of suicide deaths are by firearm injury (51%) and poisoning (14%), often by medication overdose. Lethal means safety counseling (LMSC) is a clinical intervention during which healthcare providers encourage patients to voluntarily remove lethal means (e.g., firearms) from their households or store them more safely to reduce their suicide risk (2, 3). LMSC can be delivered by a variety of providers across a range of clinical settings [e.g., emergency departments, mental health, primary care; (4, 5)], but has typically been directed towards individuals at increased risk for suicide or unintentional injury. Despite broad clinical agreement about the importance of LMSC, there is substantial variability in implementation of this evidence-based intervention across clinical settings, even among high-risk patient populations (6, 7).

Reasons for variation in delivery of LMSC are not fully understood. A systematic review of quantitative studies showed that both providers and patients, particularly those who own firearms, are hesitant to engage in LMSC, that provider training and greater perceived efficacy in counseling increase the delivery of LMSC, and that providers report offering counseling more consistently for those believed to be at higher risk of suicide or firearm injury (6). Qualitative studies are critical for explaining the individual and contextual factors that influence intervention implementation (8–10), especially for interventions about sensitive topics (e.g., firearm safety) that must address the views and concerns of a variety of stakeholders to be successful (11, 12). While qualitative studies are limited by their exploratory nature and small sample sizes, a synthesis of qualitative studies allows for the identification of themes that are consistent across multiple individual studies.

We therefore aimed to conduct a systematic review of qualitative studies to examine: (1) stakeholders’ perceptions of LMSC, especially the barriers and facilitators to its implementation, as well as the role of intervention characteristics and contextual factors on perceptions of acceptability and feasibility; (2) differences in perceptions of LMSC based on stakeholder group and clinical setting; and (3) the implications of stakeholder perceptions for informing LMSC implementation and future research directions. To help conceptualize our findings and describe ways in which stakeholder perceptions may impact the delivery and implementation of LMSC, we categorized subthemes based on domains in the Consolidated Framework for Implementation Research (CFIR), a widely used implementation framework (13).

## Methods

The protocol for this review was published on PROSPERO in March 2021, following database searching and prior to screening records for inclusion (PROSPERO ID CRD42021237515). We followed ENTREQ [Enhancing transparency in reporting the synthesis of qualitative research; (14)] and, where applicable, PRISMA reporting guidelines [Supplementary Tables 1, 2; (15)]. As we did not collect primary data, we did not seek IRB approval or patient consent.

### Search strategy

**FIGURE 1:**
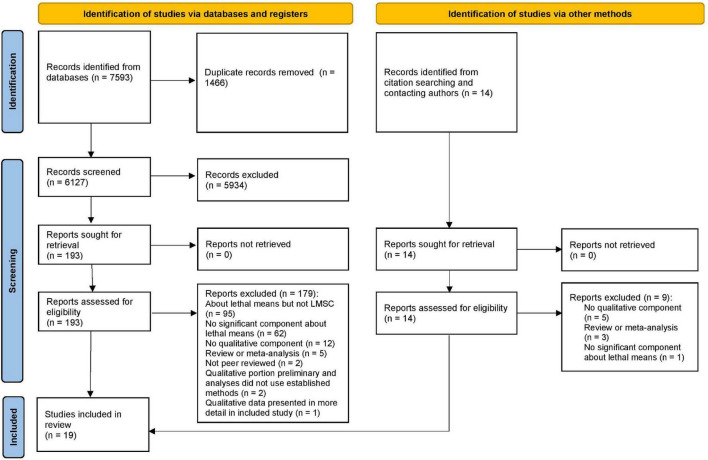
PRISMA flow diagram for study identification, screening, and inclusion.

We conducted a comprehensive search for studies by searching PubMed and PsycInfo from inception to February 2021 for terms relevant to (1) lethal means in general and firearms and medications in particular, including medications most frequently used for overdose (16); (2) suicide, safety, and injury; and (3) qualitative data (see Object S1 for full search terms). Terms for qualitative data were based on guidance from previous qualitative review papers (17, 18), compilations of relevant Medical Subject Heading (MeSH) terms, and discussions with an information scientist. Following guidance for the conduct of qualitative reviews (10), we supplemented database searches by reviewing eligible papers for relevant citations and contacting corresponding authors of eligible papers to request additional published or in-press papers on similar topics. After removing duplicates, we screened 7,593 abstracts obtained from database searching and 14 from other sources using Covidence software (Figure 1). In total, 207 full texts were reviewed. At each stage, two authors (SS, EM, or KM) reviewed each record and disagreements were resolved by a separate author (GK, during abstract screening) or consensus discussions (during full text screening).

### Inclusion and exclusion criteria

Included studies examined stakeholders’ perspectives on LMSC, defined as healthcare providers discussing with patients their access to or storage of lethal means, even if other topics were addressed. We included all types of healthcare providers and defined stakeholders as anyone impacted by LMSC, including healthcare providers and leaders, patients, patients’ family members, or members of impacted communities (e.g., firearm owners). We included studies regardless of whether the intent of LMSC was to prevent suicide, homicide, unintentional injury, or a combination of these outcomes, as the components of counseling have significant overlap across outcomes and findings from these studies are therefore informative (19). Most studies focused on LMSC for suicide risk, although several studies in pediatric settings included LMSC for unintentional injury or homicide (20–22). No exclusions were made based on participants’ demographic or clinical characteristics. As LMSC typically includes discussions about firearms and/or medications that can be used for overdose (2), we included terms relevant to these means in our searches. We also included terms describing LMSC more generally (e.g., “means safety,” “means restriction”) and did not exclude studies based on the types of lethal means addressed.

Studies needed to include qualitative assessment (e.g., interviews, focus groups) and analysis (e.g., descriptive analyses, interpretive analyses) methods (9, 23, 24). We therefore included mixed-methods studies, but excluded those in which the qualitative component consisted only of open-ended survey questions or similar data sources. According to guidelines for qualitative syntheses (25, 26), we included only peer-reviewed studies. We excluded two studies that briefly summarized preliminary qualitative data collected for another study (27, 28). Papers that were from the same parent study were marked as such (Table 1). We analyzed these as separate studies because each presented unique data and no participants overlapped. As concerns related to firearms and medications might differ, particularly due to the cultural significance of firearms in the US (29), we synthesized findings about firearms and medications separately.

**TABLE 1:** Study characteristics by year of publication.

Paper	Stakeholder groups	Context	Type of lethal mean	Assessments	Sample size	% Female	Race (% minority)	Qualitative approach
Barkin et al. (22)	Pediatricians, community leaders, and parents	Los Angeles community. Explored doctors’ roles in preventing youth injury during well-child visits.	Firearms	Interviews	26	58%	81%	Identification of themes/pile sort technique: no other info
Slovak and Singer (50)	Adolescent mental health clinicians	Rural Midwestern USA. Explored how clinicians assess for suicide risk and counsel parents on risks of firearms.	Firearms	Focus groups	24	63%	8%	Constant comparison method, inductive
Walters et al. (51)	VA facility leaders, mental health clinicians, mental health patients who currently or previously owned guns, and family members	Midwestern VA Medical Center. Explored stakeholder perceptions of firearm safety and interventions to delay firearm access.	Firearms	Focus groups and interviews	60	Patients: 0%. Family: 75%. Clinicians: 64%	NR	Iterative group process: no other info
Benjamin Wolk et al. (45)^a^	Parents, physicians, nurses, nurse practitioners, leaders of clinics, third-party payers, and members of national credentialing bodies	Midwestern and Southern health systems. Explored stakeholders’ needs related to implementing a firearm safety intervention in pediatric primary care.	Firearms	Interviews	58	53%	26%	Integrated analysis approach, deductive and inductive (grounded theory)
Gorton et al. (40)	Community pharmacy staff	North West of England, UK. Explored role of pharmacy teams in suicide prevention and limiting access to meds.	Medications	Interviews	25	72%	NR	Thematic analysis, inductive
Jager-Hyman et al. (46)^a^	Firearm safety course instructors and retailers, and law enforcement	Midwestern and Southern US regions. Explored perspectives related to implementing a firearm safety intervention in pediatric primary care.	Firearms	Interviews	12	0%	8%	Integrated approach with inductive, iterative coding
Pallin et al. (52)^b^	Firearm owners and/or people who identified as being affiliated with firearms	General population. Explored perspectives related to the development of a firearm and medication storage patient decision aid.	Firearms	Interviews	15	33%	20%	Stepwise approach, deductive and inductive
Wolf et al. (44)	Emergency department nurses	National conference of nurses in emergency departments. Assessed nurses’ perception of firearm injury risk, assessment, and counseling.	Firearms	Focus groups	25	76%	NR	Situational analysis, visual mapping: no other info
Slovak et al. (53)	Geriatric case workers	Area Agency on Aging in Ohio. Explored impact of LMSC training on case workers’ beliefs/intentions.	Firearms	Focus groups	5	80%	NR	Themes developed inductively
Aitken et al. (20)	Parents living in households with firearms	Three Southern US states with high firearm ownership. Explored parents’ firearm attitudes, beliefs, and storage practices.	Firearms	Focus groups	57	68%	14%	Grounded theory approach and comparative coding process, inductive
Monteith et al. (54)^c^	Female veterans eligible for Veterans Health services who currently or previously owned firearms	Mountain West VA Medical Center. Explored female veterans’ firearm experiences and perspectives.	Firearms	Interviews	16	100%	19%	Thematic analysis, inductive
Simonetti et al. (55)^c^	Male veterans eligible for Veterans Health services who currently or previously owned firearms	Mountain West VA Medical Center. Explored male veterans’ firearm experiences and beliefs.	Firearms	Interviews	17	0%	59%	Thematic analysis, inductive
Dobscha et al. (47)^d^	Members of local Veteran organizations, most associated with one VA Medical Center	Portland VA Medical Center. Explored veterans’ perspectives on discussing firearm safety in primary care.	Firearms	Focus groups and interviews	68	NR	NR	Grounded theory and constant comparative method, inductive
Newell et al. (48)^d^	Veterans with depression or PTSD who had recently seen a provider in primary care trained to discuss firearm safety	Portland VA Medical Center. Explored veterans’ perspectives on discussing firearm safety in primary care.	Firearms	Interviews	27	7%	33%	Hybrid conventional/directive content analysis, deductive and inductive
Hinnant et al. (21)	Pediatricians and parents	Urban, suburban, and rural Missouri. Explored perceptions of firearm safety discussions during well-child visits.	Firearms	Interviews	36	81%	33%	Constant comparison method, inductive
Salhi et al. (41)	Behavioral health clinicians treating adolescents at risk of suicide in the emergency department	Four hospital networks in Colorado. Explored impact of LMSC training on clinicians’ experiences providing LMSC.	Firearms and medications	Interviews	23	78%	NR	Constant comparison method, inductive
Richards et al. (49)	Primary care patients with suicidal thoughts	Kaiser Permanente in Washington State. Explored perspectives on being asked about access to firearms.	Firearms	Interviews	37	68%	24%	Hybrid conventional/directive content analysis, deductive and inductive
Siry et al. (42)^b^	Adults with suicidal ideation or attempts and their family members	General population. Explored experiences relevant to receiving LMSC and developing a firearm and medication storage patient decision aid.	Firearms and medications	Interviews	27	33%	11%	Thematic analysis, deductive and inductive
Siry et al. (43)^b^	Clinicians and associated staff in the emergency department	Three large emergency departments in Colorado. Explored contextual factors related to implementing a firearm and medication storage patient decision aid.	Firearms	Interviews	15	66%	NR	Thematic analysis, deductive and inductive

NR, not reported

^a–d^Papers with the same superscript were part of the same larger study. Each paper presented unique qualitative data and no participants overlapped.

### Study quality assessments

We assessed study quality using the Critical Appraisal Skills Program (30), which includes three sections evaluating the validity of results, clarity and rigor of findings, and value of the research. We also assessed comprehensiveness of reporting using the Consolidated Criteria for Reporting Qualitative Research (COREQ) checklist (31), which includes three sections evaluating reporting of research reflexivity, study design, and analysis and findings (summaries in Table 2 and details in Supplementary Tables 3–7). Two authors evaluated each study using CASP (SK and GK) and COREQ (KH and GK), and resolved disagreements through consensus discussions.

**TABLE 2:** Summary of study quality ratings by year of publication.

Paper	CASP ratings			COREQ ratings			
	Validity	Results	Value	Research team/Reflexivity (8 total)	Study design (15 total)	Analysis/Findings (9 total)	COREQ total (32 total)
Barkin et al. (22)	5 Yes 1 No	2 Yes 1 Can’t tell	1 Yes	0	9	7	16
Slovak and Singer (50)	5 Yes 1 No	2 Yes 1 No	1 Yes	0	8	7	15
Walters et al. (51)	6 Yes	2 Yes 1 Can’t tell	1 Yes	3	7	5	15
Benjamin Wolk et al. (45)^a^	5 Yes 1 No	3 Yes	1 Yes	5	11	7	23
Gorton et al. (40)	5 Yes 1 No	3 Yes	1 Yes	4	11	8	23
Jager-Hyman et al. (46)^a^	6 Yes	3 Yes	1 Yes	7	13	7	27
Pallin et al. (52)^b^	6 Yes	2 Yes 1 No	1 Yes	5	8	7	20
Wolf et al. (44)	4 Yes 2 No	2 Yes 1 No	1 Yes	0	10	3	13
Slovak et al. (53)	5 Yes 1 No	3 Yes	1 Yes	1	11	6	18
Aitken et al. (20)	4 Yes 2 No	1 Yes 1 No 1 Can’t tell	1 Yes	3	8	7	18
Monteith et al. (54)^c^	6 Yes	3 Yes	1 Yes	2	8	7	17
Simonetti et al. (55)^c^	6 Yes	3 Yes	1 Yes	1	10	6	17
Dobscha et al. (47)^d^	5 Yes 1 No	2 Yes 1 No	1 Yes	3	10	8	21
Newell et al. (48)^d^	6 Yes	3 Yes	1 Yes	0	9	7	16
Hinnant et al.,(21)	5 Yes 1 No	2 Yes 1 No	1 Yes	0	9	8	17
Salhi et al. (41)	5 Yes 1 No	3 Yes	1 Yes	3	10	8	21
Richards et al. (49)	6 Yes	3 Yes	1 Yes	3	9	7	19
Siry et al. (42)^b^	6 Yes	2 Yes 1 Can’t tell	1 Yes	2	9	9	20
Siry et al. (43)^b^	6 Yes	3 Yes	1 Yes	4	12	7	23

^a–d^Papers with the same superscript were part of the same larger study. Each paper presented unique qualitative data and no participants overlapped.

We followed recent guidelines by not basing inclusion decisions on quality ratings and instead assessing our level of confidence in the main review findings using the CERQual (Confidence in the Evidence from Reviews of Qualitative Research) Approach (8, 32). CERQual is recommended by the Cochrane Qualitative and Implementation Methods Group and involves evaluating findings according to their methodological limitations, coherence, adequacy of data, and relevance, and assigning confidence ratings based on these criteria (Table 3 and Supplementary Table 8). One author assigned confidence ratings for overarching analytic themes (GK) and another author (BH) reviewed and verified these findings; conflicts were resolved via consensus.

**TABLE 3:** Summary of findings by analytic themes and subthemes, organized by CFIR domains.

Analytic themes and subthemes	Included studies	CERQual ratings for themes and exemplar/representative quotations for subthemes
**Theme 1**: The importance of firearms to owners’ identities and perceptions of ownership as a value and right lead to perceived cultural tensions between patients and providers and hesitancy to discuss firearms.	(20, 21, 42, 44–52, 54, 55)	**High confidence:** 14 papers with no or very minor concerns about methodological limitations, coherence, adequacy, and relevance. All settings and stakeholder groups were represented.
***Characteristics of Individuals/Patient Barriers*:** Belief that firearm ownership is a protected and private right, which influences perspectives on whether providers should discuss firearms. Disclosing ownership may lead to losing one’s firearms or being tracked on a government registry.	(21, 44–50, 52, 54)	*When you just see it on this form, and you don’t know what they’re going to do about how you answer this form, for someone who is concerned about the government infringing on their rights, it gives you the feeling of, ‘Maybe I should just answer no’* [Richards et al. (49)].
Patients can feel judged by healthcare providers when being asked about firearms.	(20, 21, 45–47, 54)	*I remember just the general shock at providers when they’re like ‘Do you own firearms?’ and I said, ‘yeah.’ And they go ‘Oh my god,’ and they start looking at me weird where they’d scoot over across the room, so their behaviors, their reactions are just something that need to be worked on* [Dobscha et al. (47)].
***Characteristics of Individuals/Provider Barriers*:** Providers can be reluctant to discuss firearms due to cultural and political tensions, including fears of offending patients and their own biases about firearms.	(21, 44, 45, 51)	*I don’t want to offend a family asking the question and having them not listen to me. I try to be very careful on how […] I introduce the subject and try to keep my focus on keeping kids safe. […] there’s a lot of rhetoric out there. It can be challenging* [Hinnant et al. (21].
***Outer Setting:*** Belief that individuals have a right to protect themselves by owning and using firearms and not disclosing firearm ownership.	(21, 44, 46–52, 54, 55)	*He’s got a right to protect his family and … in my own opinion…you’ve got the gun and what happens when you’ve got your whole family there, some kook comes in, fired up on drugs like you know happens all the time and they start killing people … you lose your family because you’ve got that firing pin out of there* [Walters et al. (51)].
There is value in owning and using firearms.	(20, 21, 45, 48–50, 52, 54, 55)	*The ownership of a firearm, and I’m telling you something you already know, but it’s different than a watch.* (Pallin et al. (52)).
A perceived cultural divide impacts firearm-related discussions, which is seen as a divide between patients and providers who are often assumed to be non-owners.	(20, 21, 42, 44–46, 48, 50, 52)	*Well I think right now our country is split between those that interpret that they need to have their weapons to protect themselves and their property versus those that see the potential harm that weapons can do…* [Jager-Hyman et al. (46)]
**Theme 2**: The acceptability of LMSC, and especially asking about access, depends on understanding its rationale and context and feeling comfortable with the provider.	(20–22, 41, 43–52, 54, 55)	**High confidence:** 16 papers with no or very minor concerns about methodological limitations, coherence, adequacy, and relevance. All settings and stakeholder groups were represented.
***Characteristics of Individuals/Patient Facilitators*:** When patients understand the rationale for LMSC it increases their willingness to engage with providers.	(20, 41, 43, 48, 49)	*…your kids aren’t always going to…tell you what’s going on. So having it (the gun) out is dangerous because you might not know what’s going on with your kid, they can just kill themselves and that would be on you* [Aitken et al. (20)]
***Inner Setting:*** Stakeholders perceive LMSC as more acceptable and feasible for patients in emotional distress and parents of children and adolescents, and less so as a universal intervention.	(21, 22, 41, 44–48, 51, 55)	*In the ED I work at…when that comes up, when that question comes up, and they say they are having suicidal thoughts, we will ask, “Well, do you have a plan? Do you have any access to firearms?” Other than that, it’s not something we immediately ask* [Wolf et al. (44)].
***Intervention Characteristics:*** Providers may consider framing LMSC as part of discussions about home safety or other types of lethal means, and providing a rationale for these discussions.	(20, 21, 41, 45–50, 52, 54, 55)	*…Maybe more lead into the question, for some other people, might be less off-putting. [Not] Just, “you own firearms?” [laughs], maybe an explanation of the rationale* [Newell et al. (48)].
Patients prefer speaking about firearms with providers they trust and with whom they have an established relationship.	(22, 43, 45, 47, 50, 51, 54, 55)	*I have an amazing relationship with my [primary care provider], and if he brought it up, I believe I could be honest enough with him that… I could say I had concerns and could validate his concerns* [Monteith et al. (54)].
Stakeholders disagree whether and in which contexts to ask patients if they have access to firearms as part of LMSC.	(21, 43, 44, 46, 47, 49, 51)	*When thinking about how to change somebody’s behavior, sometimes asking if there’s a gun is almost an accusation. Instead, addressing it as if there is a gun is less of an accusation. It’s just informational. Just like if you have a dog in the house, make sure your dog isn’t rabid. You don’t have to ask if there’s a dog, but you make sure they don’t have a rabid dog* [Hinnant et al. (21)].
**Theme 3**: Cultural competency is important for discussing firearms; training providers on firearms, firearm culture, and risk for suicide can improve their competence and confidence in providing LMSC.	(20–22, 41–48, 50–53, 55)	**High confidence:** 16 papers with no or very minor concerns about methodological limitations, adequacy, and relevance, and minor concerns about coherence (specific suggestions varied across studies). All settings and stakeholder groups were represented.
***Characteristics of Individuals/Provider Barriers*:** Many providers feel that they do not have adequate understanding of firearms or firearm culture.	(21, 43, 45, 51, 53)	* Not really discomfort but just simple naiveté … it’s a totally foreign language to me* [Walters et al. (51)].
***Characteristics of Individuals/Provider Facilitators*:** Giving providers access to training on firearms and risk for suicide, as well as supporting materials, can facilitate their implementation of LMSC.	(21, 41, 43–47, 50, 51, 53)	*Absolutely… the lock box for one [thing]…Keeping the gun in one place and the ammo in another…There were quite a few techniques that were offered up that have definitely helped* [Slovak et al. (53)].
***Intervention characteristics:*** Additions to standard LMSC could include providing written information or decision tools about storage options, providing case studies or examples, and referring to community services and to organizations that provide training in firearm safety.	(20, 22, 41, 45, 46, 51–53)	*Have written materials that they can hand out…I think it would be helpful to have some scenarios where we try to anticipate what people’s responses might be* [Benjamin Wolk et al. (45)].
Providers should tailor recommendations about storage options to reasons for owning firearms, emphasize the temporary nature of changes and the range of options, and consider patients’ emotional distress.	(21, 41–43, 51, 52, 55)	*I think it should be emphasized that it is temporary and then if somebody continues to struggle, or they’re not in a position where they’re improving, then it might not be temporary…* [Pallin et al. (52)].
Providers should show cultural competence by acknowledging the role of firearms in patients’ lives, appealing to a culture of safety and responsibility, and using appropriate terminology.	(20, 21, 50, 52, 55)	*In [this town], people are really proud of their Second Amendment rights. It’s reaching into that culture and knowing where their background is* [Hinnant et al. (21)].
Providers should be nonjudgmental, respectful, and aware of their own biases; they should form a genuine connection with patients while remaining professional and impersonal about details of firearm access and storage.	(21, 45, 47, 48, 50)	*Just explaining, ‘This is why I’m asking you these questions. It’s because I care, and I don’t want to see you end up hurt.’ You know, actually showing concern instead of just like ‘Do you have—’ and reading off of a checklist* [Dobscha et al. (47)].
Patients prefer speaking about firearms with providers who own firearms or understand firearm-related values.	(20, 46, 48, 52)	*But somebody tell you [that] you need to lock your weapons up, keep your ammunition separate, making those kinds of suggestions, having some credibility might make the difference. It might make the difference between a guy taking that advice and not taking it [Pallin et al.* (52)*].*
***Process of Implementation:*** Stakeholders recommend partnering with firearm advocacy groups to support implementation of LMSC.	(45, 46)	*Why don’t we try engagement? Why don’t we try to find a way where we get on the same side of this issue, leverage our training and safety infrastructure, review the content, make sure it’s consistent with the message you’re trying to deliver, and see if in some small geography, we can lever it and study it* [Jager-Hyman et al. (46)]
**Theme 4**: Firearm owners are concerned about safety, but interpretation of safety often differs based on the individual; unsecured firearms are perceived as low risk and securing firearms can mean not having access to them when needed for defense.	(20–22, 41–43, 45, 46, 49–52, 55)	**Moderate confidence:** 13 papers with minor concerns about coherence (variation in views among firearm owners), adequacy (a few subthemes had less evidence), and relevance (not all settings fully represented). No or very minor methodological limitations.
***Characteristics of Individuals//Patient Barriers*:** Belief that the risk of unsecured firearms is low and it is important to have easy access to firearms for self-defense.	(20–22, 50, 51, 55)	*Why have it if it’s not loaded?…Safety off…Because I don’t want to sit there fumbling around if somebody comes through the window or comes through the door…* (Simonetti et al. (55))
Belief that many currently available locking devices are inconvenient, might hamper self-defense efforts, or are too expensive.	(20, 42, 46, 52)	*Most of those things didn’t work because people were like, ‘Well, you’re giving me this really clumsy thing, and I gotta find the key, and I have to hide the key or know the combination or whatever. Then I can’t get it when the burglar breaks in’* [Siry et al. (42)].
Belief that suicides and unintentional injuries are inevitable and storing firearms safely will not prevent them.	(20, 49)	*I feel like if a person really has their mind set on killing themselves, it doesn’t matter whether they have a gun or not. They will find a way* [Richards et al. (49)]
***Outer Setting:*** Safety and protection are valued by firearm owners and are reasons to have access to firearms and practice responsible ownership.	(20, 42, 46, 52, 55)	*Gun safety to me would be understanding how to use a weapon and it would be the same to me as you use a vehicle…If you really don’t know the power behind it…how to work it or if you are not familiar and you are scared to touch it, then yeah, accidents are going to happen* [Aitken et al. (20)].
***Intervention characteristics:*** Stakeholders recommend providing or subsidizing firearm locks, but disagree about the feasibility and effectiveness of funding and distributing them.	(20, 21, 41, 43, 45, 46, 51)	*There have been times we’ve had eight year olds and nine year olds that I would love to hand [lockboxes] out or…it’d be nice to still have that to offer to other people…just make it free for anybody who needs it…* [Salhi et al. (41)].
**Theme 5**: Implementing LMSC requires navigating logistical issues like provider time constraints, organization of healthcare systems, and current clinic practices.	(21, 22, 41, 43–45, 47, 51, 53)	**Moderate confidence:** 9 papers with minor concerns about coherence (specific logistical concerns varied across studies) and moderate concerns about adequacy (several subthemes had less evidence) and relevance (not all settings and stakeholders fully represented). No or very minor methodological limitations.
***Characteristics of Individuals/Provider Barriers*:** Providers are required to screen for a variety of health risks in a short time and LMSC could be an additional burden on their time.	(21, 22, 41, 43, 45, 47)	*“…staff will definitely perceive things as, ‘Oh my gosh here’s one more thing,’ like one more charting thing that we have to do and we already don’t have enough time to like get the bare minimum done”* [Siry et al. (43)].
Providers feel a lack of control over features of the healthcare system and patients’ responses to LMSC	(22, 44, 45, 51, 53)	*I just don’t know what the average life span of a member in a health maintenance organization is because if the employers were to decide to change their insurance, it may be two years or even less…* [Barkin et al. (22)].
***Characteristics of Individuals/Provider Facilitators*:** LMSC can be facilitated by integrating it into current clinic practices, including existing clinic workflows, health records, and training opportunities.	(41, 43–45, 51)	*As we beef up our training process it would be important to incorporate [LMSC] into our training and our onboarding and all of that* [Salhi et al. (41)]
***Inner Setting*:** Healthcare leaders have practical concerns about implementing LMSC, including provider time and storage infrastructure.	(45, 51)	*I don’t know what they [firearm locks] cost and I don’t think that that would necessarily be something that we would be able to invest in* [Benjamin Wolk et al. (45)]
**Theme 6**: There is value in adapting LMSC based on patients’ background and experiences.	(21, 42, 44, 45, 47–52, 54, 55)	**Moderate confidence:** 12 studies with moderate concerns about coherence (key patient subgroups not fully addressed) and adequacy (limited evidence for important subgroups) and no or very minor concerns about methodological limitations and relevance.
***Intervention Characteristics:*** Providers should adapt LMSC based on patients’ backgrounds and experiences. Specific subgroups identified include veterans, those who live in rural versus urban areas, and women.	(21, 42, 44, 45, 47–52, 54, 55)	*‘[My clients] come in with boots with blood all over them because they’ve been hunting and they’ve got hunting dogs and they’ve got guns. And guns is who they are, they are hunters, that’s who these people are. They’ve got guns, that’s not a question’* [Slovak and Singer (50)]
**Theme 7**: Family members and friends can help facilitate LMSC, but their concerns need to be addressed.	(42, 43, 46, 51, 52, 54, 55)	**Low confidence:** 7 papers with moderate concerns about relevance (primary care setting and clinicians not well represented) and coherence (views about roles/concerns varied), serious concerns about adequacy (limited evidence), and no or very minor concerns about methodological limitations.
*Characteristics of Individuals/Patient Facilitators*: Trusted family members and friends can facilitate LMSC by helping remove or store firearms and connecting the patient to care.	(42, 43, 46, 51, 52, 54, 55)	*My husband… I would probably tell him I want him to put the weapons away. To put them out of my access* [Monteith et al. (54)]
*Characteristics of Individuals/Family & Friends Barriers*: Family members may be concerned for their own safety when limiting their loved one’s access to a firearm.	(51)	*I wouldn’t be able to take the key…He would hurt me to get it* [Walters et al. (51)]

**FIGURE 2:**
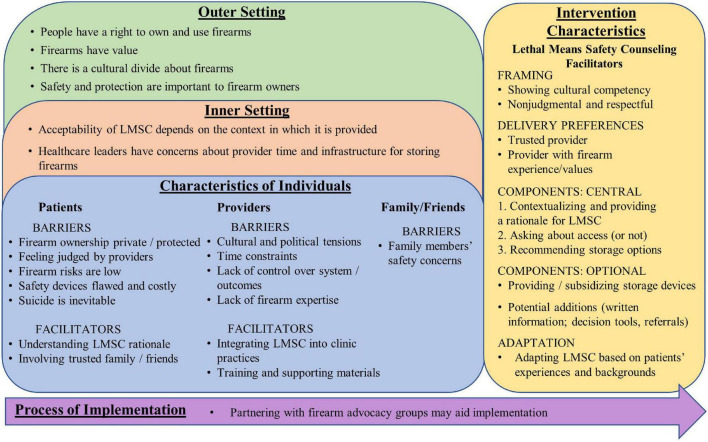
Subthemes organized by CFIR (consolidated framework for implementation research) domains.

CFIR, Consolidated framework for implementation research; CERQual, Confidence in the evidence from reviews of qualitative research. All quotes are from participants in primary papers and are not based on the authors’ interpretations. Subtheme categories are based on the CFIR – see Figure 2 for details. For more information about the CERQual ratings, see “Study Quality Assessments” and Supplementary Table 7 (CERQual Evidence Profile).

### Data extraction and analysis

Prior to analyses, the author team discussed their experiences, beliefs, and biases related to lethal means and LMSC to enhance their awareness of these factors and help manage biases during data interpretation and presentation (33). We then synthesized and interpreted findings from primary studies using thematic synthesis, an adaptation of thematic analysis for research synthesis that allows for the development of analytic themes in primary studies (9, 24, 34). Consistent with prior work (34–36), we considered the full results and discussion sections of each paper as data and imported these into NVivo. Two authors (GK and SK) reviewed all papers in detail, identified and discussed recurring concepts, developed a codebook, and coded data line-by-line using an inductive and iterative approach (9, 37). Five papers (26%) were double-coded to ensure consistency. We used coding comparisons to produce inter-rater reliability metrics that were used to understand coding discrepancies and refine the codebook. A third author (JS) also reviewed the materials and provided input on the codebook. The average kappa was 0.89 with a range of 0.71 to 1.0.

The two authors first categorized data using broad, descriptive codes such as *barriers identified by patients* and *stakeholder recommendations for increasing LMSC acceptability*. These codes were then subcategorized into detailed subthemes. Three authors (GK, KH, and JS) reviewed and consolidated these subthemes, and then organized them into overarching analytic themes. Additionally, these authors assigned subthemes to relevant CFIR domains (e.g., Intervention Characteristics) to help conceptualize ways in which stakeholder perceptions of LMSC may impact its implementation (13, 38).

After coding the qualitative data, we applied second-level codes for stakeholder group and clinical setting, as previous research suggests that there are important distinctions among these factors (6, 12, 39). For example, if a paper only reported provider perceptions it was coded as such, but if a paper reported provider and patient perceptions, passages in the paper were coded as perceptions of providers or patients, respectively. Stakeholder group codes included providers and patients, as well as firearm owners and non-owners. Clinical setting codes included emergency departments, pediatric primary care, and adult primary care. We did not code for settings with fewer than three relevant studies due to concerns about making comparisons with insufficient information (Table 1).

## Results

### Characteristics of studies

Nineteen eligible papers were identified (Figure 1), of which 16 addressed LMSC exclusively for firearms, 1 addressed LMSC exclusively for medications (40), and 2 addressed LMSC for both firearms and medications [(41, 42); Table 1]. Given the paucity of medication-related data, we briefly summarized findings on medication-related LMSC and focused our main analyses on firearm-specific LMSC.

Studies examined LMSC in the following settings (Table 1): emergency departments (41–44), pediatric primary care (21, 22, 45, 46), adult primary care (47–49), outpatient mental health (50, 51), and outside of any specific clinical setting (20, 52–55). Stakeholder groups included healthcare providers (22, 41, 43–45, 50, 51, 53), patients (20–22, 42, 45, 48, 49, 51, 54, 55), firearm owners or those with firearm expertise (20, 46, 51, 52, 54, 55), healthcare leaders (45, 51), community members (22, 47), and family members (43, 51), with many studies including more than one group or overlapping groups. Finally, five studies focused on veteran participants (47, 48, 51, 54, 55).

### Thematic synthesis

Table 3 includes summaries of overarching analytic themes and subthemes, as well as supporting quotes and the studies contributing to each theme. Figure 2 presents the subthemes grouped according to the CFIR domain to which they were assigned. The first CFIR domain, Intervention Characteristics, refers to aspects of an intervention that impact its implementation success; we assigned subthemes describing stakeholder suggestions for improving LMSC acceptability to this domain. We included subthemes relevant to the overall framing of the intervention, each intervention component, patient preferences for providers to deliver LMSC, and ways to adapt the intervention. Characteristics of Individuals (domain 2) refers to individuals’ beliefs, knowledge, personal characteristics, and values that impact implementation; subthemes describing stakeholder perceptions of barriers and facilitators to LMSC implementation were assigned to this domain. Inner Setting (domain 3), refers to characteristics of the implementation setting; subthemes describing the relevance of clinical contexts to LMSC implementation were assigned to this domain. Outer Setting (domain 4) refers to external influences on intervention implementation; subthemes describing sociopolitical beliefs about firearms were assigned to this domain. Finally, Process of Implementation (domain 5) refers to stages of implementation (e.g., planning and executing); one subtheme related to partnering with stakeholders during LMSC implementation was assigned to this domain. Subthemes within Inner Setting, Outer Setting, and Process of Implementation were not labeled as barriers or facilitators because they can be conceptualized as either depending on how they are viewed and addressed by stakeholders (56).

#### Theme 1: Perceptions of firearms as a value and right leads to a cultural divide

Firearm owners perceived firearms as being important to their identities and believed that firearms have value and that owning them is a protected and private right. These views led to perceived cultural tensions between patients and providers and hesitancy to discuss firearms. Three relevant sociopolitical beliefs about firearms were identified. First, firearms have sentimental and practical value, including for hunting, socializing, employment purposes (e.g., military service), and personal protection (20, 21, 45, 48–50, 52, 54, 55). Second, firearms ownership was viewed as justified by Constitutional rights, rights to protect oneself and one’s family, and rights to privacy, which could be jeopardized by disclosing firearm ownership to providers or limiting access to them (21, 44–52, 54). Third, patients and providers perceived a cultural divide regarding firearms, which was seen as a divide between patients who own firearms and providers who were typically assumed to be non-owners. This divide led providers and patients to hesitate to engage in LMSC (20, 21, 42, 44–46, 48–50, 52). On the other hand, providers familiar with firearms noted that referencing these experiences gave them credibility when conducing LMSC (21, 50).

The perceived cultural divide about firearms related to patient and provider barriers to engaging in LMSC. Some patients thought that providers should not be involved in firearm-related discussions and that ownership disclosure may lead to losing firearm access or government tracking (21, 44–52, 54). Patients also felt judged by providers when disclosing firearm ownership based on their perceptions of providers’ verbal responses and body language, as well as assumptions that providers were only asking certain patients about firearm safety (20, 21, 45–47, 54). Finally, providers described how cultural and political tensions surrounding firearms made it challenging to discuss patients’ safety while not offending patients or negatively impacting their relationship with them (21, 44, 45, 51).

#### Theme 2: Acceptability of lethal means safety counseling depends on its rationale and context

Patients and providers noted that the acceptability of LMSC depends on its context and rationale. They generally agreed that LMSC was acceptable and feasible for patients at elevated risk for suicide or in mental distress, and for parents of children and adolescents. Although some patients and providers thought that LMSC would be acceptable and feasible as a universal intervention for adults, others did not (21, 22, 41, 44–48, 51, 55). Patients’ comprehension of the rationale for LMSC, including protecting those vulnerable to suicides or injuries, can facilitate their engagement in it (20, 41, 43, 48, 49). Relatedly, patients prefer discussing firearms with someone they trust and with whom they have a preexisting relationship, which may be their doctor or other members of their treatment team (22, 43, 45, 47, 50, 51, 54, 55). Additionally, stakeholders noted that LMSC would be more acceptable if it included a rationale for discussing firearms, was incorporated into conversations about home safety, other health behaviors (e.g., car seats), or other lethal means (e.g., medications), and accounted for the role of other factors when appropriate (e.g., previous traumatic events) (20, 21, 41, 45–50, 52, 54, 55).

A point of disagreement was whether and in which contexts providers should ask patients if they have access to firearms (21, 43, 44, 46, 47, 49, 51). Some patients and providers preferred that providers offer advice about storing firearms without explicitly asking about firearm ownership to sidestep patients’ concerns about firearm rights and privacy, and because firearm ownership is already so common in some communities (21, 46, 47). Others noted that information about firearm access helps providers offer more targeted storage solutions and said that if these questions were asked more regularly, patients would be more willing to answer them (43, 44, 49). Firearm stakeholders in particular recommended that information about ownership not be placed in patients’ medical records (46).

#### Theme 3: Providing training to increase cultural competency

Both patients and providers highlighted the role of cultural competency in facilitating discussions about firearms, and noted that training providers to understand more about firearms, firearm culture, and suicide risk related to firearms may improve their competency and confidence in delivering LMSC. Providers perceived their lack of experience with firearms as a barrier to discussing firearm storage with patients (21, 43, 45, 51, 53). Relevant training, however, as well as supporting materials like free locking devices to give to patients, decision tools for firearm storage (e.g., Lock to Live), and written materials for patients could facilitate providers’ implementation of LMSC (21, 41, 43–47, 50, 51, 53). Additionally, firearm stakeholders recommended that health systems partner with firearm advocacy groups to provide resources that firearm owners may perceive as more credible or acceptable (45, 46).

Providers and patients also noted ways in which cultural competency can shape the delivery of LMSC. First, they recommended that providers acknowledge the value and role of firearms in people’s lives and identities, as well as appeal to the pre-existing culture of safety within the firearm community (20, 21, 50, 52, 55). Second, they noted that providers should remain non-judgmental, respectful, and aware of their own biases when discussing firearms. Specifically, providers were asked to convey their concern for the patient and try to establish a genuine connection to them, while at the same time remaining professional and impersonal about the details of firearm access and storage (21, 45, 47, 48, 50). Third, suggestions related to the recommendation of storage options included tailoring recommendations to patients’ reasons for owning firearms, offering a range of storage options to fit patients’ individual needs, emphasizing the temporary nature of limiting access to firearms during high-risk periods to encourage behavior change, and providing specific storage options to help focus individuals in emotional distress (21, 41–43, 51, 52, 55). Fourth, stakeholders made note of additional components of LMSC that may facilitate its implementation. These included providing written information to patients about firearm storage or suicide risk, describing examples of patients who stored their firearms more securely following LMSC, or referring patients to other organizations to provide them with additional services or firearm safety training (20, 22, 41, 45, 46, 51–53). Finally, patients expressed a preference for discussing firearms with a provider who owns firearms or understands values related to firearms, as they perceived these individuals to have more credibility and understanding of their situation (20, 46, 48, 52).

#### Theme 4: Beliefs about safety and risks of secured and unsecured firearms

Firearm owners generally perceived firearms and unsecured firearms as contributing little to suicide risk, and overall prioritized safety in terms of personal and household protection. In fact, securing firearms increased the chance of not having access to them when needed for personal or household protection. Firearm owners stated that their valuation of safety motivated them both to have easy access to firearms for protection and to practice responsible ownership (20, 42, 45, 46, 51, 52, 55). Responsible ownership included preventing unauthorized access to firearms and practicing safe handling (46, 52, 55), as well as introducing firearms to children and teaching them to respect firearms and handle them safely (20, 46). These practices were for the purposes of preventing unintentional injury rather than suicide.

Beliefs about safety and risk were related to several patient barriers to LMSC. First, patients perceived the risk of unsecured firearms in terms of suicide or unintentional injury as low compared to other safety risks (e.g., access to alcohol, risk of victimization) for themselves and their families, and prioritized easy access to their firearms for self-defense (20–22, 50, 51, 55). Second, some patients expressed the belief that storing firearms safely would not prevent suicides or unintentional injuries as such incidents are inevitable (20, 49). Third, patients noted that conventional locks, particularly trigger and cable locks, were inconvenient to use and might hamper self-defense efforts. Some reported that these locks were often unused or disposed of when they were provided (20, 42, 46, 52). Although biometric storage devices were perceived as less problematic, their cost was a deterrent (20). These views led patients and providers to recommend that locking devices be provided or subsidized by the healthcare system, but there were conflicting opinions about the feasibility of funding and distributing them, whether patients would use them, and their effectiveness in preventing suicide (20, 21, 41, 43, 45, 46, 51).

#### Theme 5: Logistical concerns about implementing lethal means safety counseling

Providers, healthcare leaders, and patients reported logistical concerns about implementing LMSC, including provider time constraints, the organization of the healthcare system, and current clinic practices. Providers and patients noted that providers’ time constraints and competing demands serve as barriers to conducting LMSC, particularly if counseling is not brief (21, 22, 41, 43, 45, 47). Providers also described features of the healthcare system and patients’ responses to LMSC that are beyond their control, including limited resources for referrals to mental health care, the potential for legal implications if patients decide not to secure firearms or do so incorrectly, and not being able to ensure that patients followed through on securing firearms (22, 44, 45, 51, 53). Healthcare leaders also noted practical barriers to implementing LMSC, including limited provider time, lack of funding and infrastructure for storing firearms, and clinician turnover (45, 51). On the other hand, providers highlighted potential facilitators to LMSC implementation, including integrating it into clinic workflows, using electronic medical records to prompt providers and monitor implementation, adding provider training to ongoing training opportunities, and having providers aside from doctors (e.g., nurses, medical assistants) deliver LMSC (41, 43–45, 51).

#### Theme 6: Adapting lethal means safety counseling to individual patients

Patients and providers indicated that LMSC should be adapted to patients’ backgrounds and experiences, including the framing and rationale, asking about firearm access, and recommending storage options (21, 42, 44, 45, 47–52, 54, 55). Relevant patient characteristics included veterans, individuals who live in rural versus urban settings, and women. Veterans wanted providers to acknowledge their expertise with firearms, and those familiar with veterans noted that their camaraderie with one another may make it easier for them to trust other veterans when discussing firearms (42, 47, 48, 51, 52, 54, 55). Patients and providers stated that in some rural communities, firearm ownership is very common, firearms are often used for hunting, and both privacy and the right to own firearms are highly valued (21, 44, 45, 50, 55). In urban settings, on the other hand, some patients may be more likely to own firearms for personal protection (44, 45). A study of female veterans found that many women were familiarized to firearms via men in their lives (54), and another noted that not all patients realize that firearms are a common method of suicide among women (50).

#### Theme 7: Family members and friends can facilitate lethal means safety counseling

Patients noted that when family members, friends, or others (e.g., a fellow veteran) are trusted, these individuals can facilitate LMSC by helping to remove or store firearms, connecting the patient to care, or initiating conversations about firearm access when the patient is at risk for suicide (42, 43, 46, 51, 52, 54, 55). On the other hand, some family members expressed concern for their own safety when they are put in charge of limiting their loved one’s access to a firearm (51).

### Differences across stakeholder groups and clinical settings

When examining differences in subthemes based on stakeholder groups, we found that some subthemes were raised more often by patients than providers. Specifically, patients noted that safety and protection are valued by firearm owners in multiple studies (20, 42, 51, 52, 55), while this was only briefly acknowledged by providers in one study (45). By contrast, the cultural divide regarding firearm-related discussions was raised by providers and patients alike across multiple studies (20, 21, 43–45, 48, 50). Additionally, while patients noted concerns about the quality and cost of firearm storage devices (20, 42), providers did not mention these concerns. We were unable to analyze differences between firearm owners and non-owners, as many studies did not reliably differentiate between these groups. We were also unable to formally analyze differences among various clinical settings as there were insufficient data in each category (emergency departments, adult primary care, and pediatric primary care). We did, however, explore topics specific to pediatric versus adult primary care settings to generate ideas for future research. We found that providers and parents interviewed in the context of pediatric primary care settings were concerned about unintentional injury resulting from youth gaining access to firearms in addition to suicide risk, while those in adult primary care settings focused on suicide risk alone (20, 21, 45, 46). Additionally, as noted in Theme 2, stakeholders in pediatric primary care settings highlighted the value of providers offering advice on safe storage of firearms to all patients as a universal intervention (20, 21, 45) to a greater extent than stakeholders in adult primary care settings, although this view was present to some extent in adult primary care settings as well (47–49).

### Medication-related lethal means safety counseling

One paper that examined the potential contributions of community pharmacy teams for suicide prevention in the United Kingdom focused only on medication-related LMSC (40), and two papers that examined both medications and firearms did so within the context of understanding the impact of LMSC training (41) and the development of a firearm and medication storage decision aid (42). The first study (40) highlighted the potential role of pharmacists embedded in the community in identifying patients at risk for suicide and referring them for additional care. While pharmacists rarely mentioned their role in limiting the amount of medication disbursed to at-risk patients, the authors note that further research is needed to examine this issue. Another study (41) described how training in LMSC, as well as the ability to provide medication lockboxes, encouraged providers to counsel parents to remove or lock up medications in the home to limit the risk of overdose among adolescents with behavioral health problems. Finally, the last study (42) noted that decision aids could help patients in the emergency room or other contexts decide how best to store their medications.

## Discussion

Our systematic review yielded 19 studies examining stakeholder perceptions of LMSC and its implementation using qualitative methodology. The 18 papers on LMSC related to firearms included a variety of settings (emergency departments, pediatric and adult primary care, and outpatient mental health) and stakeholders (providers, patients, members of the firearm community, healthcare leaders, and community and family members). We identified seven overarching themes that described the meaning and value of firearms to owners’ identities, their views of firearm ownership as a right, and the implications of these views for perceived cultural tensions between patients and providers. While firearm owners were concerned about safety, they were not typically concerned about the risks of unsecured firearms. Additionally, the context in which LMSC was provided and providers’ cultural competency was seen as critical to discussing firearms. Stakeholder recommendations included addressing logistical barriers to LMSC implementation, adapting LMSC to patients’ background and preferences, and potentially involving trusted family members or friends.

Previous reviews have highlighted providers and patients’ hesitancy to engage in LMSC and the importance of provider training, in addition to examining the efficacy of LMSC in changing storage behavior and the ways it is delivered in practice (6, 57–60). As the first review to synthesize findings from qualitative studies on LMSC, the themes we identified provide a more nuanced analysis of the individual and contextual factors that impact LMSC implementation. Analyzing multiple studies also enabled us to identify themes common across different settings and stakeholder groups, and to overcome some of the limitations of individual qualitative studies like small sample sizes. These themes inform clinical, operational and research endeavors aiming to increase the reach and effectiveness of firearm counseling. More specifically, they provide guidance on characteristics of the intervention that may increase acceptability, barriers and facilitators to implementation, and the role of both the clinical setting and larger sociopolitical contexts relevant to discussions about firearm access and storage.

Our review identified several gaps in the LMSC literature that need to be addressed in future research. First, perspectives of various stakeholders, like patients’ family and friends and healthcare leaders, as well as subgroups of individuals (e.g., based on gender, veteran status, geographical location, race/ethnicity, socioeconomic status), were underrepresented in the extant literature. Second, we were unable to differentiate between certain individual characteristics (e.g., firearm owners and non-owners) because studies did not clearly identify participants as such, and the few studies in each clinical setting precluded our analysis of differences across these settings. Third, we found only three studies that explored stakeholders’ perspectives on LMSC for medications and no studies on LMSC for means aside from firearms and medications (e.g., rope). Furthering research in this area is critical, as about half of suicides in the US are not related to firearms. Additionally, rates of death related to poisoning and overdose have risen sharply in recent years, particularly during the COVID-19 pandemic (61). New psychoactive substances like synthetic opioids, which are linked to higher rates of overdose and suicide risk on their own and in combination with medications like benzodiazepines, are also growing in popularity (61, 62). Further qualitative research on LMSC specific to medications and recreational drugs is therefore especially critical at this time. Fourth, only one theme was relevant to the CFIR domain of Process of Implementation and two themes were relevant to Inner Setting, highlighting the limited research relevant to these areas. Notably, the majority of papers included in our review (15 out of 19) were published since 2019, consistent with an uptick in firearm-related research funding and publication nationally (63) and suggesting that some of the gaps in the literature may soon be addressed [e.g., (64)].

This review should be considered alongside several study limitations. First, although the suggestions stemming from this work inform the delivery and implementation of LMSC, most of them have yet to be empirically tested with respect to their acceptability, feasibility, and efficacy. For example, stakeholders reported that emphasizing that changes to firearm storage may be temporary can improve LMSC acceptability. However, we do not know whether temporary changes to storage are effective in preventing suicide nor whether using this language leads to increases in LMSC acceptability. Second, we excluded quantitative studies on LMSC and studies on LMSC effectiveness – while other recent reviews present this research (6, 57–60), these exclusions still limit the scope of our paper. Third, a number of papers we identified were conducted by the same group of investigators within the same parent study – a total of nine published papers stemmed from four parent studies (Table 1). While we analyzed these papers separately because they each included unique groups of participants, this trend further highlights the limited research in this area. Fourth, although we followed guidelines for qualitative syntheses by including only peer-reviewed studies (25, 26), this may have resulted in the exclusion of pertinent studies described in dissertations or other non-peer-reviewed sources. Fifth, some studies in pediatric settings included LMSC for unintentional injury or homicide as well as suicide (20–22) and the extent to which these specific injury outcomes impact perspectives on firearm interventions is unclear. Finally, as in all qualitative syntheses, we aimed to describe a large number of qualitative studies providing in-depth and nuanced information, and were unable to include all relevant data from each study. Therefore, the scope of this synthesis is necessarily constrained to the information we chose to present.

In sum, this study highlights important new findings in the field of LMSC, a rapidly growing field in which understanding and addressing stakeholders’ perceptions is particularly critical (11, 65). Future research should explore the perspectives of patients’ family and friends, healthcare leaders, and subgroups of patients from diverse sociodemographic backgrounds and clinical settings. Additional research is also needed to formally evaluate ways in which stakeholders’ suggestions outlined here impact LMSC effectiveness and implementation.

## Data availability statement

The original contributions presented in this study are included in the article/Supplementary material, further inquiries can be directed to the corresponding author.

## Author contributions

GK led the study, including study conceptualization, data analysis, and manuscript writing. GK, SK, KH, and JS participated in data analysis. GK, KH, SS, and EM participated in literature reviewing. All authors contributed to manuscript revision, and read and approved the final version.
