# Supplementary material for: Stakeholder perceptions of lethal means safety counseling: A qualitative systematic review
Source: Front Psychiatry. 2022 Oct 20;13:993415. doi: 10.3389/fpsyt.2022.993415 (PMC9634731; doi:10.3389/fpsyt.2022.993415)
Supplement: Supplementary file 1 [file Data_Sheet_1.docx]

**Object S1.** Full search strings

Pubmed

(“lethal means”[TIAB] OR “means safety”[TIAB] OR “means counseling”[TIAB] OR “means restriction”[TIAB] OR firearm*[TIAB] OR gun[TIAB] OR guns[TIAB] OR medication*[TIAB] OR drug*[TIAB] OR opiate*[TIAB] OR benzodiazepine*[TIAB] OR pill[TIAB] OR pills[TIAB] OR poison*[TIAB])

AND

(Suicid*[TIAB] OR safety[TIAB] OR injur*[TIAB])

AND

("Qualitative Research"[MeSH] OR “Interviews as Topic”[MeSH] OR “Focus Groups”[MeSH] OR “Grounded Theory”[MeSH] OR “Nursing Methodology Research”[MESH] OR qualitative[TIAB] OR interview*[TIAB] OR “focus group*”[TIAB] OR “grounded theory”[TIAB] OR phenomenolog*[TIAB])

PsycInfo

(AB(“lethal means” OR “means safety” OR “means counseling” OR “means restriction” OR firearm* OR gun OR guns OR medication* OR drug* OR opiate* OR benzodiazepine* OR pill OR pills OR poison*) OR TI(“lethal means” OR “means safety” OR “means counseling” OR “means restriction” OR firearm* OR gun OR guns OR medication* OR drug* OR opiate* OR benzodiazepine* OR pill OR pills OR poison*) OR KW(“lethal means” OR “means safety” OR “means counseling” OR “means restriction” OR firearm* OR gun OR guns OR medication* OR drug* OR opiate* OR benzodiazepine* OR pill OR pills OR poison*))

AND

(AB(Suicid* OR safety OR injur*) OR TI(Suicid* OR safety OR injur*) OR KW(Suicid* OR safety OR injur*))

AND

(AB(qualitative OR interview* OR “focus group*” OR “grounded theory” OR phenomenolog*) OR TI(qualitative OR interview* OR “focus group*” OR “grounded theory” OR phenomenolog*) OR KW(qualitative OR interview* OR “focus group*” OR “grounded theory” OR phenomenolog*))

*Note:* In both databases, we restricted search results to English language studies. Searches were conducted in February 2021.

**Table S1**. ENTREQ (Enhancing transparency in reporting the synthesis of qualitative research) checklist

| **No. Item** | **Guide Questions/Description** | **Where Reported** |
| --- | --- | --- |
| 1. Aim | State the research question the synthesis addresses | Page 3 |
| 2. Synthesis methodology | Identify the synthesis methodology or theoretical framework which underpins the synthesis, and describe the rationale for choice of methodology (e.g. metaethnography, thematic synthesis, critical interpretive synthesis, grounded theory synthesis, realist synthesis, meta-aggregation, meta-study, framework synthesis). | Page 5 |
| 3. Approach to searching | Indicate whether the search was pre-planned (comprehensive search strategies to seek all available studies) or iterative (to seek all available concepts until theoretical saturation is achieved). | Page 4 |
| 4. Inclusion criteria | Specify the inclusion/exclusion criteria (e.g. in terms of population, language, year limits, type of publication, study type). | Page 4 |
| 5. Data sources | Describe the information sources used (e.g. electronic databases (MEDLINE, EMBASE, CINAHL, psychINFO, Econlit), grey literature databases (digital thesis, policy reports), relevant organisational websites, experts, information specialists, generic web searches (Google Scholar), hand searching, reference lists) and when the searches were conducted; provide the rationale for using the data sources. | Page 4 |
| 6. Electronic Search strategy | Describe the literature search (e.g. provide electronic search strategies with population terms, clinical or health topic terms, experiential or social phenomena related terms, filters for qualitative research and search limits). | Page 4 and Object S1 |
| 7. Study screening methods | Describe the process of study screening and sifting (e.g. title, abstract and full text review, number of independent reviewers who screened studies) | Page 4 |
| 8. Study characteristics | Present the characteristics of the included studies (e.g. year of publication, country, population, number of participants, data collection, methodology, analysis, research questions). | Table 1 |
| 9. Study selection results | Identify the number of studies screened and provide reasons for study exclusion (e.g. for comprehensive searching, provide numbers of studies screened and reasons for exclusion indicated in a figure/flowchart; for iterative searching describe reasons for study exclusion and inclusion based on modifications to the research question and/or contribution to theory development). | Page 4 and Figure 1 |
| 10. Rationale for appraisal | Describe the rationale and approach used to appraise the included studies or selected findings (e.g. assessment of conduct (validity and robustness), assessment of reporting (transparency), assessment of content and utility of the findings). | Pages 4-5 |
| 11. Appraisal items | State the tools, frameworks and criteria used to appraise the studies or selected findings (e.g. Existing tools: CASP, QARI, COREQ, Mays and Pope [25]; reviewer developed tools; describe the domains assessed: research team, study design, data analysis and interpretations, reporting). | Pages 4-5 (CASP, COREQ, CERQual) |
| 12. Appraisal process | Indicate whether the appraisal was conducted independently by more than one reviewer and if consensus was required. | Pages 4-5 |
| 13. Appraisal results | Present results of the quality assessment and indicate which articles, if any, were weighted/excluded based on the assessment and give the rationale. | Tables 2-3 and S3-S8 |
| 14. Data extraction | Indicate which sections of the primary studies were analysed and how were the data extracted from the primary studies? (e.g. all text under the headings “results /conclusions” were extracted electronically and entered into a computer software). | Page 5 |
| 15. Software | State the computer software used, if any | Page 5 (NVivo) |
| 16. Number of reviewers | Identify who was involved in coding and analysis. | Page 5 |
| 17. Coding | Describe the process for coding of data (e.g. line by line coding to search for concepts) | Page 5 |
| 18. Study comparison | Describe how were comparisons made within and across studies (e.g. subsequent studies were coded into pre-existing concepts, and new concepts were created when deemed necessary) | Page 5 |
| 19. Derivation of themes | Explain whether the process of deriving the themes or constructs was inductive or deductive. | Page 5 |
| 20. Quotations | Provide quotations from the primary studies to illustrate themes/constructs, and identify whether the quotations were participant quotations or the author’s interpretation | Table 3 |
| 21. Synthesis output | Present rich, compelling and useful results that go beyond a summary of the primary studies (e.g. new interpretation, models of evidence, conceptual models, analytical framework, development of a new theory or construct). | Pages 6-10, Table 3, and Figure 2 |

**Table S2**. PRISMA (Preferred Reporting Items for Systematic reviews and Meta-Analyses) checklist

| **Section and Topic** | **Item #** | **Checklist item** | **Location where item is reported** |
| --- | --- | --- | --- |
| **TITLE** | | |  |
| Title | 1 | Identify the report as a systematic review. | Title |
| **ABSTRACT** | | |  |
| Abstract | 2 | See the PRISMA 2020 for Abstracts checklist. | Abstract |
| **INTRODUCTION** | | |  |
| Rationale | 3 | Describe the rationale for the review in the context of existing knowledge. | Page 3 (Intro) |
| Objectives | 4 | Provide an explicit statement of the objective(s) or question(s) the review addresses. | Page 3 (Intro) |
| **METHODS** | | |  |
| Eligibility criteria | 5 | Specify the inclusion and exclusion criteria for the review and how studies were grouped for the syntheses. | Page 4 (Inclusion/Exclusion) |
| Information sources | 6 | Specify all databases, registers, websites, organisations, reference lists and other sources searched or consulted to identify studies. Specify the date when each source was last searched or consulted. | Page 4 (Search Strategy) |
| Search strategy | 7 | Present the full search strategies for all databases, registers and websites, including any filters and limits used. | Page 4 (Search Strategy); Object S1 |
| Selection process | 8 | Specify the methods used to decide whether a study met the inclusion criteria of the review, including how many reviewers screened each record and each report retrieved, whether they worked independently, and if applicable, details of automation tools used in the process. | Page 4 (Search Strategy) |
| Data collection process | 9 | Specify the methods used to collect data from reports, including how many reviewers collected data from each report, whether they worked independently, any processes for obtaining or confirming data from study investigators, and if applicable, details of automation tools used in the process. | Page 5 (Data Extraction and Analysis) |
| Data items | 10a | List and define all outcomes for which data were sought. Specify whether all results that were compatible with each outcome domain in each study were sought (e.g. for all measures, time points, analyses), and if not, the methods used to decide which results to collect. | NA |
|  | 10b | List and define all other variables for which data were sought (e.g. participant and intervention characteristics, funding sources). Describe any assumptions made about any missing or unclear information. | NA |
| Study risk of bias assessment | 11 | Specify the methods used to assess risk of bias in the included studies, including details of the tool(s) used, how many reviewers assessed each study and whether they worked independently, and if applicable, details of automation tools used in the process. | Pages 4-5 (Study Quality Assessments) |
| Effect measures | 12 | Specify for each outcome the effect measure(s) (e.g. risk ratio, mean difference) used in the synthesis or presentation of results. | NA |
| Synthesis methods | 13a | Describe the processes used to decide which studies were eligible for each synthesis (e.g. tabulating the study intervention characteristics and comparing against the planned groups for each synthesis (item #5)). | NA |
|  | 13b | Describe any methods required to prepare the data for presentation or synthesis, such as handling of missing summary statistics, or data conversions. | NA |
|  | 13c | Describe any methods used to tabulate or visually display results of individual studies and syntheses. | NA |
|  | 13d | Describe any methods used to synthesize results and provide a rationale for the choice(s). If meta-analysis was performed, describe the model(s), method(s) to identify the presence and extent of statistical heterogeneity, and software package(s) used. | Page 5 (Data Extraction and Analysis) |
|  | 13e | Describe any methods used to explore possible causes of heterogeneity among study results (e.g. subgroup analysis, meta-regression). | Page 5 (Data Extraction and Analysis) |
|  | 13f | Describe any sensitivity analyses conducted to assess robustness of the synthesized results. | NA |
| Reporting bias assessment | 14 | Describe any methods used to assess risk of bias due to missing results in a synthesis (arising from reporting biases). | NA |
| Certainty assessment | 15 | Describe any methods used to assess certainty (or confidence) in the body of evidence for an outcome. | Page 5 (Study Quality Assessments) |
| **RESULTS** | | |  |
| Study selection | 16a | Describe the results of the search and selection process, from the number of records identified in the search to the number of studies included in the review, ideally using a flow diagram. | Figure 1 |
|  | 16b | Cite studies that might appear to meet the inclusion criteria, but which were excluded, and explain why they were excluded. | Page 4 (Inclusion/Exclusion) |
| Study characteristics | 17 | Cite each included study and present its characteristics. | Table 1 |
| Risk of bias in studies | 18 | Present assessments of risk of bias for each included study. | Tables 2 and S3-S7 |
| Results of individual studies | 19 | For all outcomes, present, for each study: (a) summary statistics for each group (where appropriate) and (b) an effect estimate and its precision (e.g. confidence/credible interval), ideally using structured tables or plots. | NA |
| Results of syntheses | 20a | For each synthesis, briefly summarise the characteristics and risk of bias among contributing studies. | Tables 1-3 |
|  | 20b | Present results of all statistical syntheses conducted. If meta-analysis was done, present for each the summary estimate and its precision (e.g. confidence/credible interval) and measures of statistical heterogeneity. If comparing groups, describe the direction of the effect. | NA |
|  | 20c | Present results of all investigations of possible causes of heterogeneity among study results. | NA |
|  | 20d | Present results of all sensitivity analyses conducted to assess the robustness of the synthesized results. | NA |
| Reporting biases | 21 | Present assessments of risk of bias due to missing results (arising from reporting biases) for each synthesis assessed. | NA |
| Certainty of evidence | 22 | Present assessments of certainty (or confidence) in the body of evidence for each outcome assessed. | Tables 3 and S8 |
| **DISCUSSION** | | |  |
| Discussion | 23a | Provide a general interpretation of the results in the context of other evidence. | Pages 10-11 |
|  | 23b | Discuss any limitations of the evidence included in the review. | Pages 11-12 |
|  | 23c | Discuss any limitations of the review processes used. | Page 11 |
|  | 23d | Discuss implications of the results for practice, policy, and future research. | Page 11 |
| **OTHER INFORMATION** | | |  |
| Registration and protocol | 24a | Provide registration information for the review, including register name and registration number, or state that the review was not registered. | Page 3 (Methods) |
|  | 24b | Indicate where the review protocol can be accessed, or state that a protocol was not prepared. | Page 3 |
|  | 24c | Describe and explain any amendments to information provided at registration or in the protocol. | NA |
| Support | 25 | Describe sources of financial or non-financial support for the review, and the role of the funders or sponsors in the review. | Title page |
| Competing interests | 26 | Declare any competing interests of review authors. | Title page |
| Availability of data, code and other materials | 27 | Report which of the following are publicly available and where they can be found: template data collection forms; data extracted from included studies; data used for all analyses; analytic code; any other materials used in the review. | NA |

*Note:* Some items are marked “NA” because they do not apply to systematic reviews of qualitative studies.

**Table S3**. CASP (Critical Appraisal Skills Program) Checklist

| **Authors** | **Year** | Aims | Method | Research Design | Sampling | Data Collection | Reflexivity | Ethical Issues | Data Analysis | Findings | Value of Research |
| --- | --- | --- | --- | --- | --- | --- | --- | --- | --- | --- | --- |
| Barkin et al. | 1999 | Y | Y | Y | Y | Y | N | ? | Y | Y | Y |
| Slovak & Singer | 2012 | Y | Y | Y | Y | Y | N | Y | N | Y | Y |
| Walters et al. | 2012 | Y | Y | Y | Y | Y | Y | ? | Y | Y | Y |
| Wolk et al.^a^ | 2018 | Y | Y | Y | Y | Y | N | Y | Y | Y | Y |
| Gorton et al. | 2019 | Y | Y | Y | Y | Y | N | Y | Y | Y | Y |
| Jager-Hyman et al.^a^ | 2019 | Y | Y | Y | Y | Y | Y | Y | Y | Y | Y |
| Pallin et al. | 2019 | Y | Y | Y | Y | Y | Y | Y | N | Y | Y |
| Wolf et al. | 2019 | Y | Y | Y | N | Y | N | Y | N | Y | Y |
| Slovak et al. | 2019 | Y | Y | Y | Y | Y | N | Y | Y | Y | Y |
| Aitken et al. | 2020 | Y | Y | Y | Y | N | N | N | ? | Y | Y |
| Monteith et al.^b^ | 2020 | Y | Y | Y | Y | Y | Y | Y | Y | Y | Y |
| Simonetti et al.^b^ | 2020 | Y | Y | Y | Y | Y | Y | Y | Y | Y | Y |
| Dobscha et al.^c^ | 2021 | Y | Y | Y | Y | N | Y | N | Y | Y | Y |
| Newell et al.^c^ | 2021 | Y | Y | Y | Y | Y | Y | Y | Y | Y | Y |
| Hinnant et al. | 2021 | Y | Y | Y | Y | Y | N | N | Y | Y | Y |
| Salhi et al. | 2021 | Y | Y | Y | Y | Y | N | Y | Y | Y | Y |
| Richards et al. | 2021 | Y | Y | Y | Y | Y | Y | Y | Y | Y | Y |
| Siry, Knoepke et al.^d^ | 2021 | Y | Y | Y | Y | Y | Y | Y | ? | Y | Y |
| Siry, Polzer et al.^d^ | 2021 | Y | Y | Y | Y | Y | Y | Y | Y | Y | Y |
| Note. Ratings represent answers to questions in the CASP Checklist: Y = Yes; N = No, ? = Can't Tell. | | | | | | | | | | |  |
| ^a-d^ Papers with the same superscript were part of the same larger study. Each paper presented unique qualitative data and no participants overlapped. | | | | | | | | | |  |  |

**Table S4**. COREQ (Consolidated Criteria for Reporting Quantitative Studies) Ratings, Domain 1 (Research Team and Reflexivity)

| **Domain 1. Research Team and Reflexivity** | | **1. Interviewer/ facilitator** | **2. Credentials** | **3. Occupation** | **4. Gender** | **5. Experience and training** | **6. Relationship with participants established** | **7. Participant knowledge of interviewer** | **8. Interviewer characteristics** | **Total (out of 8)** |
| --- | --- | --- | --- | --- | --- | --- | --- | --- | --- | --- |
| Barkin et al. | 1999 | No | No | No | No | No | No | No | No | 0 |
| Slovak & Singer | 2012 | No | No | No | No | No | No | No | No | 0 |
| Walters et al. | 2012 | No | No | 693 | No | 693 | No | 693 | No | 3 |
| Wolk et al.^a^ | 2018 | No | 5 | 4 | 5 | No | 4 | 4 | No | 5 |
| Gorton et al. | 2019 | 4 | 4 | 4 | No | 4 | No | No | No | 4 |
| Jager-Hyman et al.^a^ | 2019 | 693 | 693 | 693 | 693 | 693 | 693 | 693 | No | 7 |
| Pallin et al. | 2019 | 3 | 3 | 3 | No | 3 | 3 | No | No | 5 |
| Wolf et al. | 2019 | No | No | No | No | No | No | No | No | 0 |
| Slovak et al. | 2019 | 54 | No | No | No | No | No | No | No | 1 |
| Aitken et al. | 2020 | 470 | No | No | No | 470 | No | 471 | No | 3 |
| Monteith et al.^b^ | 2020 | No | 1730 | 1730 | No | No | No | No | No | 2 |
| Simonetti et al.^b^ | 2020 | 5 | No | No | No | No | No | No | No | 1 |
| Dobscha et al.^c^ | 2021 | 2 | 2 | 2 | No | No | No | No | No | 3 |
| Newell et al.^c^ | 2021 | No | No | No | No | No | No | No | No | 0 |
| Hinnant et al. | 2021 | No | No | No | No | No | No | No | No | 0 |
| Salhi et al. | 2021 | 2 | 2 | No | No | 2 | No | No | No | 3 |
| Richards et al. | 2021 | 2 | 2 | 2 | No | No | No | No | No | 3 |
| Siry, Knoepke et al.^d^ | 2021 | No | No | 472 | No | 472 | No | No | No | 2 |
| Siry, Polzer et al.^d^ | 2021 | 96 | No | 96 | No | 96 | Appendix A | No | No | 4 |
| *Note.* Ratings represent the items in the first domain of the COREQ checklist, applied to each study in the review | | | | | | | | | |  |
| ^a-d^ Papers with the same superscript were part of the same larger study. Each paper presented unique qualitative data and no participants overlapped. | | | | | | | | | |  |

**Table S5**. COREQ (Consolidated Criteria for Reporting Quantitative Studies) Ratings, Domain 2 (Study Design) Part I

| **Domain 2. Study Design** | | **9. Methodological orientation** | **10. Sampling** | **11. Method of approach** | **12. Sample size** | **13. Non-participation** | **14. Setting** | **15. Presence of non-participants** |
| --- | --- | --- | --- | --- | --- | --- | --- | --- |
| Barkin et al. | 1999 | 54 | 54 | 54 | 54 | 54 | No | No |
| Slovak & Singer | 2012 | 214 | 214 | 214 | 214 | No | No | No |
| Walters et al. | 2012 | 694 | 693 | 693 | 694 | No | No | No |
| Wolk et al.^a^ | 2018 | 4 | 3 | 3 | 4 | No | No | 4 |
| Gorton et al. | 2019 | 4 | 3 | 3 | 4 | No | 3 | 3 |
| Jager-Hyman et al.^a^ | 2019 | 694 | 693 | 693 | 693 | 693 | No | 693 |
| Pallin et al. | 2019 | 3 | No | 3 | 3 | No | No | No |
| Wolf et al. | 2019 | 59 | No | No | 57 | No | 57 | No |
| Slovak et al. | 2019 | 52 | 53 | 53 | 52 | 53 | 54 | No |
| Aitken et al. | 2020 | 471 | 471 | 471 | 471 | No | No | No |
| Monteith et al.^b^ | 2020 | 1739 | 1738 | 1738 | 1739 | No | 1738 | No |
| Simonetti et al.^b^ | 2020 | 5 | 3 | 3 | 3 | 3 | No | No |
| Dobscha et al.^c^ | 2021 | 6 | 2 | 2 | 2 | No | 6 | No |
| Newell et al.^c^ | 2021 | 340 | 340 | 340 | 340 | 340 | No | No |
| Hinnant et al. | 2021 | 4 | 4 | 4 | 4 | No | No | No |
| Salhi et al. | 2021 | 3 | 2 | 2 | 4 | 4 | No | No |
| Richards et al. | 2021 | 2 | 2 | 2 | 3 | No | No | No |
| Siry, Knoepke et al.^d^ | 2021 | 473 | 472 | 472 | 473 | No | No | No |
| Siry, Polzer et al.^d^ | 2021 | 96 | 96 | 96 | 96 | Appendix A | No | Appendix A |
| *Note.* Ratings represent the items in the second domain of the COREQ checklist, applied to each study in the review | | | | | | | | |
| ^a-d^ Papers with the same superscript were part of the same larger study. Each paper presented unique qualitative data and no participants overlapped. | | | | | | | | |

**Table S6**. COREQ (Consolidated Criteria for Reporting Quantitative Studies) Ratings, Domain 2 (Study Design) Part 2

| **Domain 2. Study Design** | | **16. Description of sample** | **17. Interview guide** | **18. Repeat interviews** | **19. Recording** | **20. Field notes** | **21. Duration** | **22. Data Saturation** | **23. Transcripts returned** | **Total (out of 15)** |
| --- | --- | --- | --- | --- | --- | --- | --- | --- | --- | --- |
| Barkin et al. | 1999 | 54 | No | No | 54 | No | 54 | 54 | No | 9 |
| Slovak & Singer | 2012 | 214 | 214 | 220 | 214 | No | No | No | No | 8 |
| Walters et al. | 2012 | 694 | No | No | 694 | No | 694 | No | No | 7 |
| Wolk et al.^a^ | 2018 | 5 | No | 4 | 4 | 4 | 4 | 4 | 4 | 11 |
| Gorton et al. | 2019 | 4 | S1 File | No | 3 | No | 3 | 4 | No | 11 |
| Jager-Hyman et al.^a^ | 2019 | 695 | 694 | 693 | 694 | 694 | 694 | 694 | No | 13 |
| Pallin et al. | 2019 | 3 | No | No | 3 | 3 | 3 | 8 | No | 8 |
| Wolf et al. | 2019 | 60 | 57 | No | 59 | 59 | 57 | 59 | 59 | 10 |
| Slovak et al. | 2019 | 52 | 53 | 52 | 52 | 54 | No | No | No | 11 |
| Aitken et al. | 2020 | 471 | No | No | 471 | 471 | No | 471 | No | 8 |
| Monteith et al.^b^ | 2020 | 1740 | No | No | 1738 | No | No | 1739 | No | 8 |
| Simonetti et al.^b^ | 2020 | 6 | Appendix | No | 4 | No | 3 | 5 | No | 10 |
| Dobscha et al.^c^ | 2021 | 2 | 3 | 2 | 2 | No | 2 | No | No | 10 |
| Newell et al.^c^ | 2021 | 340 | 340 | No | 340 | No | 340 | No | No | 9 |
| Hinnant et al. | 2021 | 4 and Appendix A | Appendix B | No | 4 | No | 4 | 4 | No | 9 |
| Salhi et al. | 2021 | 4 | 3 | No | 2 | No | 3 | 3 | No | 10 |
| Richards et al. | 2021 | 3 | 2 | No | 2 | No | 3 | 2 | No | 9 |
| Siry, Knoepke et al.^d^ | 2021 | 473 | No | No | 472 | 472 | 472 | 473 | No | 9 |
| Siry, Polzer et al.^d^ | 2021 | 96 | No | Appendix A | 96 | No | 96 | 96 | Appendix A | 12 |
| *Note.* Ratings represent the items in the second domain of the COREQ checklist, applied to each study in the review | | | | | | | | | |  |
| ^a-d^ Papers with the same superscript were part of the same larger study. Each paper presented unique qualitative data and no participants overlapped. | | | | | | | | |  |  |

**Table S7**. COREQ (Consolidated Criteria for Reporting Quantitative Studies) Ratings, Domain 3 (Analysis and Findings)

| **Domain 3. Analysis and Findings** | | **24. Number of coders** | **25. Description of coding tree** | **26. Derivation of themes** | **27. Software** | **28. Participant checking** | **29. Quotes presented** | **30. Data and findings consistent** | **31. Clarity of major themes** | **32. Clarity of minor themes** | **Total (out of 9)** | **COREQ total (out of 32)** |
| --- | --- | --- | --- | --- | --- | --- | --- | --- | --- | --- | --- | --- |
| Barkin et al. | 1999 | 54 | 55 | 54 | N | N | Y (55-56) | Y (55-57) | Y (55-56) | Y (55-56) | 7 | 16 |
| Slovak & Singer | 2012 | 214 | N | 214 | N | 220 | Y (214-217) | Y (214-220) | Y (214-217) | Y (214-217) | 7 | 15 |
| Walters et al. | 2012 | 694 | 694 | N | N | N | Y (694-697) | Y (694-698) | Y (694-697) | N | 5 | 15 |
| Wolk et al.^a^ | 2018 | 4 | N | N | 4 | 4 | Y (Table 3) | Y (5-9) | Y (5-9) | Y (5-9) | 7 | 23 |
| Gorton et al. | 2019 | 4 | 4 | 4 | 4 | N | Y (5-11) | Y (5-15) | Y (5-11) | Y (5-11) | 8 | 23 |
| Jager-Hyman et al.^a^ | 2019 | 695 | N | 694 | 694 | N | Y (696-697) | Y (695-699) | Y (696-697) | Y (696-697) | 7 | 27 |
| Pallin et al. | 2019 | 3 | N | 4 | 3 | N | Y (4-7 and Table 2) | Y (4-9 and Table 2) | Y (4-7 and Table 2) | Y (4-7 and Table 2) | 7 | 20 |
| Wolf et al. | 2019 | N | N | N | N | 59 | Y (61-64) | Y (61-65) | NA | NA | 3 | 13 |
| Slovak et al. | 2019 | 52 | N | 52 | 52 | N | Y (56-60) | Y (56-63) | Y (56-60) | N | 6 | 18 |
| Aitken et al. | 2020 | 471 | N | 471 | 471 | N | Y (472-474) | Y (472-476) | Y (472-474) | Y (472-474) | 7 | 18 |
| Monteith et al.^b^ | 2020 | 1739 | 1739 | 1739 | N | N | Y (1741-1748) | Y (1741-1750) | Y (1741-1748) | Y (1741-1748) | 7 | 17 |
| Simonetti et al.^b^ | 2020 | 5 | N | 5 | N | N | Y (5-12) | Y (5-15) | Y (5-12) | Y (5-12) | 6 | 17 |
| Dobscha et al.^c^ | 2021 | 6 | 6 | 6 | 6 | N | Y (6-8) | Y (6-10) | Y (6-8) | Y (6-8) | 8 | 21 |
| Newell et al.^c^ | 2021 | 340 | N | 340 | 340 | N | Y (341-343) | Y (341-343) | Y (341-343) | Y (341-343) | 7 | 16 |
| Hinnant et al. | 2021 | 4 | 4 | 4 | 4 | N | Y (5 to 8) | Y (5 to 10) | Y (5 to 8) | Y (5 to 8) | 8 | 17 |
| Salhi et al. | 2021 | 2 | 3 | 3 | 3 | N | Y (4 to 7) | Y (4 to 8) | Y (4 to 7) | Y (4 to 7) | 8 | 21 |
| Richards et al. | 2021 | 2 | Online supplement | 3 | 2 | N | Y (3-5) | Y (3-6) | Y (3-5) | Y (3-5) | 7 | 19 |
| Siry, Knoepke et al.^d^ | 2021 | 472 | 473 | 473 | 473 | 473 | Y (473-475) | Y (473-476) | Y (473-475) | Y (473-475) | 9 | 20 |
| Siry, Polzer et al.^d^ | 2021 | 96 | N | 96 | 96 | Appendix A | Y (96-98) | Y (96-99) | Y (96-98) | Y (96-98) | 7 | 23 |
| Note. Ratings represent the items in the third domain of the COREQ checklist, applied to each study in the review | | | | | | | | | | | | |
| ^a-d^ Papers with the same superscript were part of the same larger study. Each paper presented unique qualitative data and no participants overlapped. | | | | | | | | | |  |  |  |

**Table S8.** CERQual (Confidence in the Evidence from Reviews of Qualitative Research) Evidence Profile

| **Review finding** | **Methodological limitations** | **Coherence** | **Adequacy of data** | **Relevance** |
| --- | --- | --- | --- | --- |
| **Theme 1 (14 papers)**: The importance of firearms to owners’ identities and perceptions of ownership as a protected and private right lead to cultural tensions between patients and providers that decreases their willingness to discuss firearms. | No or very minor concerns. Only 2/14 papers had more than minor methodological limitations. | No or very minor concerns. Patients and providers explicitly discussed rights/values related to firearms, perceived cultural tensions, and how these factors decreased their willingness to discuss firearms. | No or very minor concerns. | No or very minor concerns. 11 papers with direct relevance, 2 with partial relevance, and 1 with indirect relevance. All types of settings and stakeholder groups were represented. |
| **Theme 2 (16 papers)**: The acceptability of LMSC, and especially asking about access, depends on understanding its rationale and context and feeling comfortable with the provider delivering it. | No or very minor concerns. Only 2/16 papers had more than minor methodological limitations. | No or very minor concerns. Stakeholders agreed that the acceptability of LMSC depends on the contextual factors noted. | No or very minor concerns. | No or very minor concerns. 12 papers with direct relevance, 3 with partial relevance, and 1 with indirect relevance. All types of settings and stakeholder groups were represented. |
| **Theme 3 (16 papers)**: Cultural competence is key to discussing firearms; training providers on firearms, firearm culture, and risk for suicide can improve their competence and confidence in providing LMSC and can shape its delivery. | No or very minor concerns. Only 2/16 papers had more than minor methodological limitations. | Minor concerns. Stakeholders agreed on the importance of cultural competence and training providers. While related suggestions for improving LMSC acceptability varied somewhat across papers, similar topics were raised. | No or very minor concerns. | No or very minor concerns. 10 papers with direct relevance, 4 with partial relevance, and 2 with indirect relevance. All types of settings and stakeholder groups were represented. |
| **Theme 4 (13 papers)**: Firearm owners are concerned about safety; unsecured firearms are perceived as low risk and securing firearms can mean not having access to them when needed. | No or very minor concerns. Only 1/13 papers had more than minor methodological limitations. | Minor concerns: While this finding represents the views of many firearm owners, there were exceptions to these perspectives as well. | Minor concerns: A few subthemes were mentioned by only a few papers or were discussed briefly. | Minor concerns. 9 papers with direct relevance, 2 with partial relevance, and 2 with indirect relevance. Most settings were included, but there was only 1 paper about adult primary care. All stakeholder groups were represented. |
| **Theme 5 (9 papers)**: Implementing LMSC requires navigating logistical issues like provider time constraints, organization of healthcare systems, and current clinic practices. | No or very minor concerns. Only 1/9 papers had more than minor methodological limitations. | Minor concerns: While logistical concerns were mentioned consistently across papers, the specific concerns varied. As this finding was descriptive and focused on logistical issues more generally, these concerns remained minor. | Moderate concerns: Several subthemes were mentioned by only a few papers or were discussed briefly. | Moderate concerns: 5 papers with direct relevance, 3 with partial relevance, and 1 with indirect relevance. Most settings were included, but there was only 1 paper about adult primary care. Views of healthcare leaders were poorly represented. |
| **Theme 6 (12 papers)**: There is value in adapting LMSC based on patients’ background and experiences. | No or very minor concerns. Only 1/12 papers had more than minor methodological limitations. | Moderate concerns. Stakeholders agreed that adapting LMSC is valuable, but key patient subgroups to consider and ways of adapting LMSC for each subgroup were not fully addressed. | Moderate concerns: Papers on each subgroup were limited and patients from minoritized backgrounds were not considered. | No or very minor concerns. 9 papers with direct relevance, 2 with partial relevance, and 1 with indirect relevance. All settings and stakeholder groups were represented. |
| **Theme 7 (7 papers)**: Family members and friends can help facilitate LMSC, but their concerns need to be addressed. | No or very minor concerns. Papers only had minor methodological limitations. | Moderate concerns. There was agreement that trusted family members and friends could facilitate LMSC, but views about their roles and barriers to their involvement varied across stakeholders. | Serious concerns: One of two subthemes was derived from only one paper. | Moderate concerns. 5 papers with direct relevance, 1 with partial relevance, and 1 with indirect relevance. Adult primary care not represented and pediatric primary care was represented by only one paper. Clinicians not well represented among stakeholders. |

*Note*. For more information about the CERQual approach, see “Study Quality Assessments” and Lewin et al., 2018.
